# Natural product derivative BIO promotes recovery after myocardial infarction via unique modulation of the cardiac microenvironment

**DOI:** 10.1038/srep30726

**Published:** 2016-08-11

**Authors:** Yong Sook Kim, Hye-yun Jeong, Ah Ra Kim, Woong-Hee Kim, Haaglim Cho, JungIn Um, Youngha Seo, Wan Seok Kang, Suk-Won Jin, Min Chul Kim, Yong-Chul Kim, Da-Woon Jung, Darren R. Williams, Youngkeun Ahn

**Affiliations:** 1 Biomedical Research Institute, Chonnam National University Hospital, Gwangju, 61469, Republic of Korea; 2 Research Laboratory of Cardiovascular Regeneration, Chonnam National University Hospital, Gwangju, 61469, Republic of Korea; 3 School of Life Sciences, Gwangju Institute of Science and Technology, 1 Oryong-Dong, Buk-Gu, Gwangju 61005, Republic of Korea; 4 Department of Cardiology, Chonnam National University Hospital, Gwangju 61469, Republic of Korea

## Abstract

The cardiac microenvironment includes cardiomyocytes, fibroblasts and macrophages, which regulate remodeling after myocardial infarction (MI). Targeting this microenvironment is a novel therapeutic approach for MI. We found that the natural compound derivative, BIO ((2′Z,3′E)-6-Bromoindirubin-3′-oxime) modulated the cardiac microenvironment to exert a therapeutic effect on MI. Using a series of co-culture studies, BIO induced proliferation in cardiomyocytes and inhibited proliferation in cardiac fibroblasts. BIO produced multiple anti-fibrotic effects in cardiac fibroblasts. In macrophages, BIO inhibited the expression of pro-inflammatory factors. Significantly, BIO modulated the molecular crosstalk between cardiac fibroblasts and differentiating macrophages to induce polarization to the anti-inflammatory M2 phenotype. In the optically transparent zebrafish-based heart failure model, BIO induced cardiomyocyte proliferation and completely recovered survival rate. BIO is a known glycogen synthase kinase-3β inhibitor, but these effects could not be recapitulated using the classical inhibitor, lithium chloride; indicating novel therapeutic effects of BIO. We identified the mechanism of BIO as differential modulation of p27 protein expression and potent induction of anti-inflammatory interleukin-10. In a rat MI model, BIO reduced fibrosis and improved cardiac performance. Histological analysis revealed modulation of the cardiac microenvironment by BIO, with increased presence of anti-inflammatory M2 macrophages. Our results demonstrate that BIO produces unique effects in the cardiac microenvironment to promote recovery post-MI.

Myocardial infarction (MI) is a leading cause of death, irrespective of socioeconomic status and ethnicity^1^. Medical interventions have been developed to stabilize patients with MI, which has improved survival rates, but there is currently no clinically approved method to reverse the loss of cardiac muscle (cardiomyocytes). Patients that survive acute MI can develop heart failure because of remodeling, due to the poor capacity of heart regeneration^2^.

The small molecule compound, BIO ((2′Z,3′E)-6-Bromoindirubin-3′-oxime) is a cell permeable derivative of the natural product, 6-bromoindirubin^3^. This compound is produced by predatory rock snails, such as *Hexaplex trunculus*, and has been used since ancient times to produce the famous ‘Tyrian purple’ dye^3^. BIO was shown to inhibit the multifunctional enzyme, glycogen synthase kinase-3β (GSK-3β). This inhibition reduced β-catenin phosphorylation and activated the Wnt signaling pathway, which maintains the undifferentiated state of stem cells^4^. Interestingly, it was shown that BIO treatment could induce proliferation in post-mitotic adult rat cardiomyocytes^5^. However, to our knowledge, BIO has not been tested as a drug that can modulate cardiac remodeling to improve heart function after MI.

The cardiac cellular composition of the microenvironment post-MI comprises: cardiomyocytes, cardiac fibroblasts, macrophages, endothelial cells and smooth muscle cells^6^. Cardiomyocytes account for only 30–40% of the total cell population in the heart^7^. Non-cardiomyocyte cell types carry out important roles to maintain cardiac homeostasis. For example, cardiac fibroblasts are the predominant cell type in terms of cell number and are located alongside cardiomyocytes throughout the heart^8^. Cardiac fibroblasts maintain myocardial architecture, contribute to cardiac electrophysiology, and communicate with cardiomyocytes via gap junctions. In pathological states, fibroblasts form a coupled network to regulate the inflammatory response, nutrient/metabolite homeostasis and spread of the infarcted region^9^. Moreover, fibroblast-cardiomyocyte networks communicate the signals from infarcted cardiomyocytes to produce a wave of cell death that spreads beyond the initial site of cardiac damage^10^.

Cardiac macrophages are another significant cell type in the cardiac microenvironment and carry out pivotal regulatory functions during remodeling after MI^11^. Cardiac macrophages can be broadly classified into two phenotypes: 1) M1, which are pro-inflammatory and remove debris from dead cells, and 2) M2, which are anti-inflammatory and promote wound resolution. M1 and M2 macrophages can be distinguished using specific markers, such as aginase-1 (Arg), inducible nitric oxide synthase (iNOS) and interleukin-10 (IL-10)^12^. Generally, M1 macrophages are iNOS^high^ Arg^low^ and M2 macrophages are iNOS^low^ Arg^high^. After MI, circulating monocytes are recruited to the infarction and converted to macrophages by signaling through cytokine receptors, such as chemokine receptor 2^13^. Studies have shown that increasing the numbers of M2 macrophages improved cardiac recovery^14^.

In this study, we evaluated the effect of the BIO on the cardiac microenvironment. Interestingly, we observed that BIO induces opposing effects in cardiomyocytes and cardiac fibroblasts, which could promote cardiac functional recovery during remodeling. Significantly, BIO can shift macrophages from an M1 to M2 phenotype. These positive effects on cardiac recovery were validated in a zebrafish cardiomyocyte depletion system and confirmed in a rat MI model. The effects of BIO were characterized as regulation of IL-10 expression and modulation of p27. Our results indicate a significant reappraisal of the biological activity of BIO in the context of cardiac remodeling after MI and establish this compound as an attractive candidate for drug development.

## Results

### BIO Selectively Induces Cardiomyocyte Proliferation and Blocks Cardiac Fibroblast Proliferation

**Figure 1:**
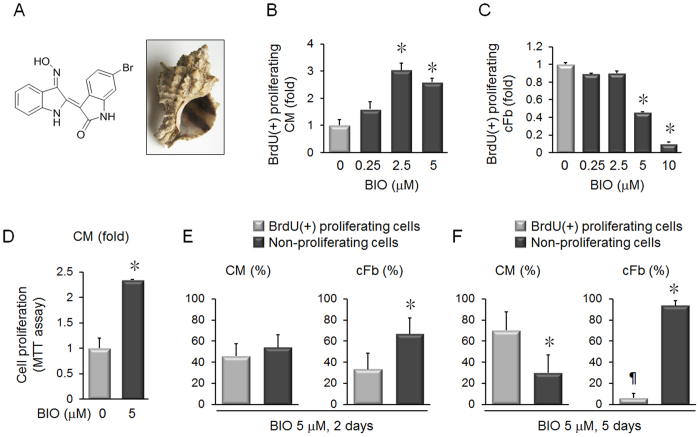
BIO preferentially induces proliferation in cardiomyocytes but not cardiac fibroblasts. (**A**) Chemical structure of BIO and photograph of *Hexaplex trunculus*. The image (https://upload.wikimedia.org/wikipedia/commons/thumb/c/c8/Mu%C5%A1le.jpg/1024px-Mu%C5%A1le.jpg) is obtained from Wikipedia and used under the Creative Commons Attribution-Share Alike 3.0 International license (http://creativecommons.org/licenses/by/3.0/). (**B**) Neonatal rat cardiomyocytes were treated with BIO for 5 days and cardiac troponin I(+)BrdU(+) proliferating cardiomyocytes were counted. BIO treatment increased proliferation in cardiomyocytes. n = 5, *p < 0.05, *t*-test. (**C**) Neonatal rat cardiac fibroblasts were treated with BIO for 5 days and cardiac troponin I(−)BrdU(+) proliferating cardiac fibroblasts were counted. BIO treatment significantly decreased proliferation in cardiac fibroblasts. n = 5, *p < 0.05, *t*-test. (**D**) The increase in cardiomyocyte proliferation after treatment with BIO for 4 days was also confirmed by MTT assay. (**E**) The numbers of proliferating cardiomyocytes and cardiac fibroblasts were determined by immunofluorescence staining with BrdU, and 5 μM BIO treatment of cardiomyocyte: cardiac fibroblast co-cultures for 2 days did not affect the proportions of proliferating and non-proliferating cells. n = 4, *p < 0.05, *t*-test. (**F**) The numbers of proliferating cardiomyocytes and cardiac fibroblasts were determined by immunofluorescence staining with BrdU, and 5 μM BIO treatment of co-cultures for 5 days produced a significant increase in the proportion of proliferating cardiomyocytes compared to non-proliferating cardiomyocytes. BIO also induced a significant reduction in the proportion of proliferating cardiac fibroblasts compared to non-proliferating fibroblasts. n = 4, *p < 0.05 vs. proliferating cardiomyocytes; ^¶^*p* < 0.05 vs. non-proliferating cardiac fibroblasts, *t*-test.

The chemical structure of BIO and the snail, *Muricidae,* is shown in Fig. 1A. Primary neonatal rat cardiomyocytes were treated with BIO for 5 days and showed a significant increase in proliferation (Fig. 1B). In contrast, BIO treatment reduced neonatal rat cardiac fibroblast proliferation (Fig. 1C). The increased proliferation of cardiomyocytes treated with BIO for 4 days was also confirmed by MTT assay (Fig. 1D). To model the cardiac microenvironment, co-cultures of cardiomyocytes and cardiac fibroblasts were treated with BIO, which increased cardiomyocyte proliferation and inhibited cardiac fibroblast proliferation (Fig. 1E,F).

### BIO Inhibits Pro-fibrotic Mechanisms

**Figure 2:**
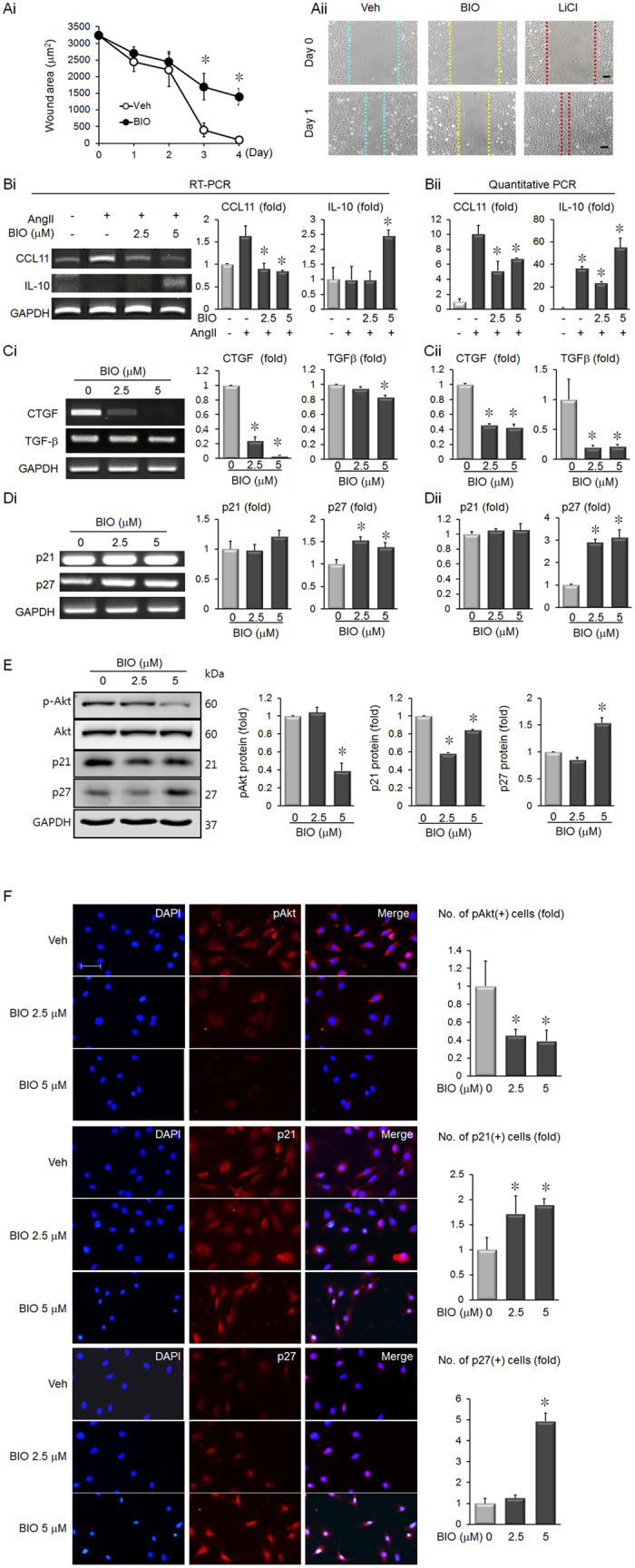
BIO inhibits cardiac fibroblast motility, negatively regulates pro-fibrotic factors, reduces Akt activity and increases p27 expression. (**Ai**) Measurement of scratch area closure indicates that BIO treatment reduced neonatal rat cardiac fibroblast motility. n = 3, *p < 0.05, *t*-test. (**Aii**) Treatment with 5 μM BIO inhibited cardiac fibroblast motility. Untreated fibroblasts invaded the scratched area to a greater extent than BIO treated fibroblasts. In contrast, fibroblasts treated with 20 mM LiCl showed greater migration compared to untreated cardiac fibroblasts. Scale bar = 100 μm. n = 3, *p < 0.05, *t*-test. (**Bi**) BIO treatment for 3 days decreased mRNA expressions of the pro-fibrotic CCL11 and the anti-fibrotic IL-10 in 100 nM AngII-activated cardiac fibroblasts. Densitometry analyses were expressed as graphs. n = 3, *p < 0.05, *t*-test. (**Bii**) Quantitative PCR was performed to confirm the levels of CCL11 mRNA and IL-10 mRNA in AngII-activated cardiac fibroblasts. (**Ci**) BIO treatment for 3 days decreased mRNA expressions of the pro-fibrotic cytokines CTGF and TGF-β in cardiac fibroblasts. Densitometry analyses were expressed as graphs. n = 3, *p < 0.05, *t*-test. (**Cii**) Quantitative PCR was performed to confirm the levels of CTGF mRNA and TGF-β mRNA. *p < 0.05, *t*-test. (**Di**) BIO did not affect p21 mRNA expression and reduced p27 mRNA expression in cardiac fibroblasts. Cells were treated with BIO for 3 days. Densitometry analyses were expressed as graphs. n = 3, *p < 0.05, *t*-test. (**Dii**) Quantitative PCR was performed to confirm the levels of p21 mRNA and p27 mRNA in AngII-activated cardiac fibroblasts. *p < 0.05, *t*-test. (**E**) In BIO treatment of cardiac fibroblasts for 1 day, the protein levels of phosphorylated Akt, p21, and p27 were assessed. Densitometry analyses were expressed as graphs. n = 3, *p < 0.05, t-test. (**F**) Protein expressions of pAkt, p21 and p27 were determined by immunofluorescence staining in cardiac fibroblasts treated with BIO for 1 day. *p < 0.05, *t*-test. Scale bar = 100 μm.

The effect of BIO on cardiac fibroblast motility was assessed using the scratch assay. BIO treatment inhibited fibroblast motility (Fig. 2Ai). BIO is used as a GSK-3β inhibitor and LiCl is one of the most widely used GSK-3β inhibitors^15^. However, LiCl did not reduce fibroblast motility (Fig. 2Aii).

In consequence of the scratch test results, the effect of BIO on fibrotic factors was assessed. BIO treatment blocked pro-fibrotic CCL11 upregulation in AngII-stimulated cardiac fibroblasts (Fig. 2Bi,Bii). BIO also increased anti-fibrotic IL-10 expression in AngII-stimulated cardiac fibroblasts (Fig. 2Bi,Bii). In addition, the expression of pro-fibrotic factors CTGF and TGF-β was reduced by BIO treatment (Fig. 2Ci,Cii).

### BIO Increases p27 Expression and Reduces Akt Signaling

To investigate the effect of BIO on proliferation in cardiac fibroblasts, immunoblotting was carried out for known regulators. BIO increased expression of the cell cycle inhibitor, p27 (Fig. 2Di,Dii). BIO treatment also increased p27 mRNA levels, but did not affect p21 mRNA (Fig. 2Di,Dii). In contrast, treatment with BIO decreased the phosphorylation of Akt, which regulates PI3K/Akt/mTOR signaling, and reduced expression of the cell cycle inhibitor, p21 (Fig. 2E). Immunofluorescence staining also confirmed that phosphorylated Akt and p21 were significantly reduced, while p27 was increased in BIO-treated cardiac fibroblasts (Fig. 2F).

### BIO Modulates Macrophage Polarization

**Figure 3:**
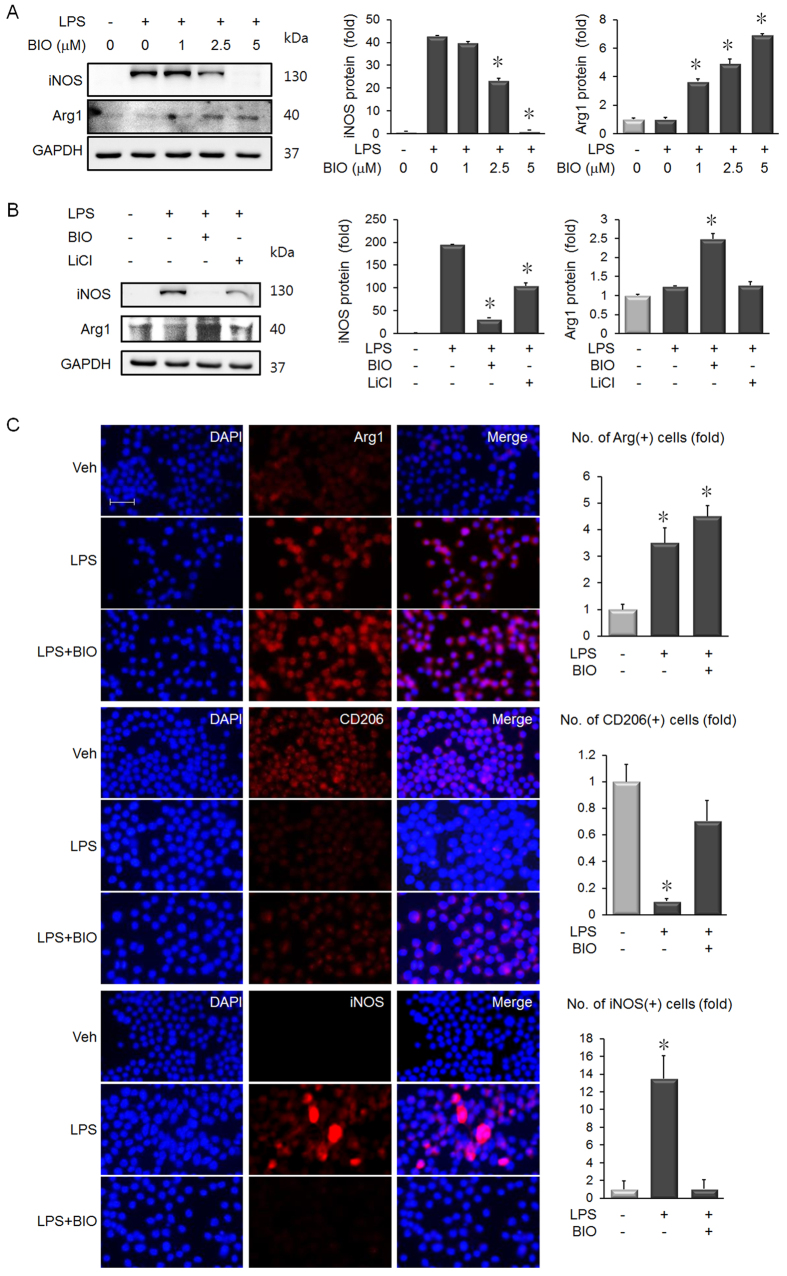
BIO reduces expression of the pro-inflammatory M1 macrophage marker, iNOS. (**A**) LPS (100 ng/mL) treatment for 24 hours induced expression of iNOS in RAW 264.7 macrophages. BIO inhibited iNOS induction in a concentration-dependent manner, and densitometry analysis of iNOS and Arg1 was expressed as a graph. n = 3, *p < 0.05, *t*-test. (**B**) Treatment of RAW 264.7 macrophages with 5 μM BIO is more effective than 20 mM LiCl at inhibiting iNOS induction by LPS. Densitometry analysis of iNOS and Arg1 expression. n = 3, *p < 0.05, *t*-test. (**C**) Immunofluorescence staing of Arg1, CD206 and iNOS expression in the treated macrophages. *p < 0.05, *t*-test. Scale bar = 100 μm.

Murine macrophage cell line RAW264.7 were stimulated with lipopolysaccharide (LPS) to induce pro-inflammatory M1 polarization, which is measured by increased expression of iNOS. Treatment with BIO blocked iNOS expression, and induced expression of the M2 anti-inflammatory macrophage marker, arginase-1 (Arg1) (Fig. 3A). In contrast, LiCl (20 mM) was less effective than BIO (5 μM) at reducing iNOS induction. Arg1 expression was also increased by BIO treatment, but not by LiCl treatment (Fig. 3B). Immunofluorescence staining demonstrated upregulation of Arg1 and recovered expression of the M2 marker, CD206. LPS-stimulated iNOS induction was also blocked by BIO treatment (Fig. 3C).

**Figure 4:**
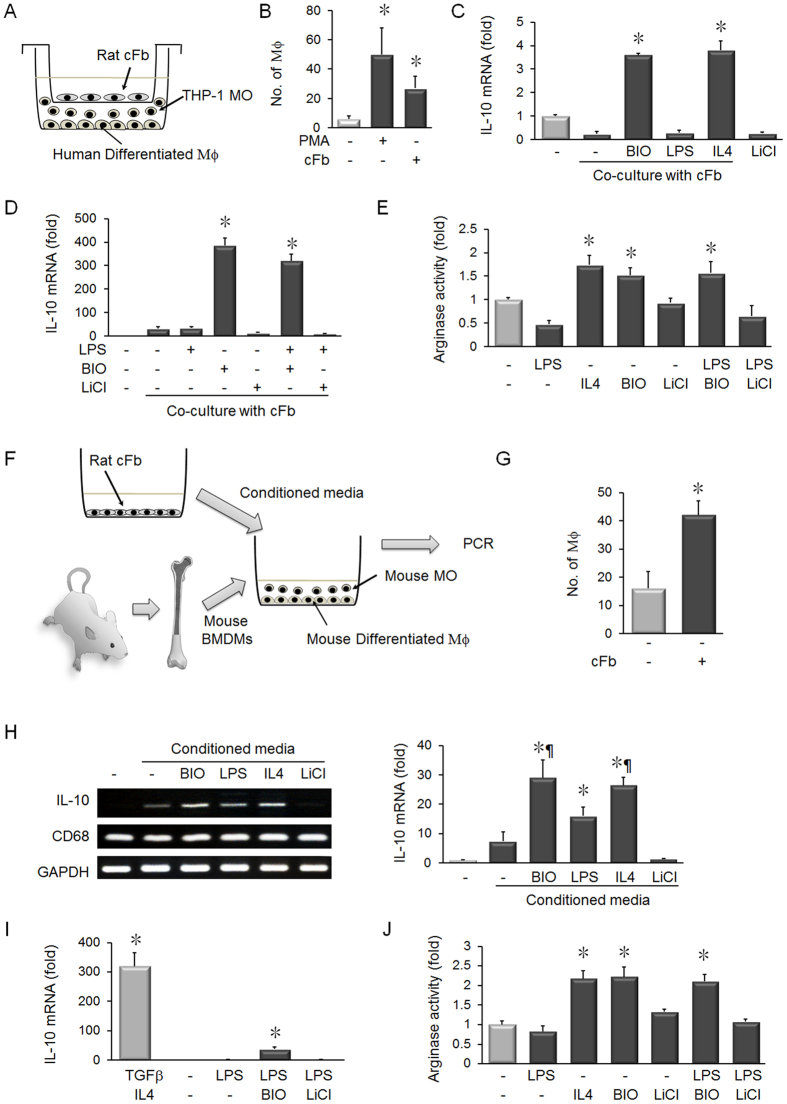
BIO induces anti-inflammatory M2 macrophage polarization in differentiating monocytes. (**A**) Schematic of the co-culture system used to analyze the influence of rat cardiac fibroblasts on human THP-1 monocyte differentiation into macrophages. (**B**) Co-culture of cardiac fibroblasts with THP-1 monocytes for 72 hours induced macrophage differentiation. Treatment with 200 nM PMA was used as a positive control for inducing macrophage differentiation. n = 4, *p < 0.05 vs. monocultures, *t*-test. (**C**) Addition of 5 μM BIO or 10 ng/mL IL-4 to the co-cultures induced IL-10 expression. n = 4, *p < 0.05, *t*-test. (**D**) Treatment of co-cultures with BIO induced the expression of IL-10 mRNA. n = 4, *p < 0.05 compared to untreated co-cultures, *t*-test. (**E**) Treatment of co-cultures with BIO or IL-4 increased Arg activity. Treatment with LiCl did not increase Arg activity. n = 4, *p < 0.05, *t*-test. (**F**) Schematic of the *ex vivo* system used to analyze the effect of BIO treatment on macrophage differentiation in mouse bone marrow-derived monocytes (BMDMs). The images of the mouse and bone are used under the Creative Commons Licenses (CC BY-SA 2.0 FR and CC BY-SA 3.0; bone image citation: “Blausen gallery 2014”. Wikiversity Journal of Medicine. DOI: 10.15347/wjm/2014.010. ISSN 20018762; mouse image citations: David Liao and User:Rama). (**G**) Conditioned media (CM) from rat cardiac fibroblasts can induce macrophage differentiation in BMDMs after for 72 h. n = 4, *p < 0.05, *t*-test. (**H**) Differentiating BMDMs treated with cardiac fibroblast CM and BIO showed increased expression of IL-10 mRNA. Treatment with LiCl did not increase IL-10, whereas treatment with IL-4 or 10 ng/mL LPS increased IL-10 expression. Densitometry analysis indicated that treatment with BIO or IL-4 induced IL-10 expression to a greater degree than BMDMs treated with LPS or CM alone. n = 4, *p < 0.05 vs. BMDMs treated with cardiac fibroblast CM, ¶*p* < 0.05 vs. BMDMs treated with CM and LPS, *t*-test. (**I**) Real-time PCR analysis indicated that treatment of BMDMs with BIO increased the expression of IL-10. As a positive control for M2 macrophage polarization, BMDMs were treated with 50 ng/mL TGF-β and 10 ng/mL IL-4 for 72 hours. n = 4, *p < 0.05 vs. BMDM treated with LiCl and LPS; ¶*p* < 0.05 vs. BMDMs differentiated in the presence of LPS, *t*-test. (**J**) Treatment of differentiation BMDMs with BIO or IL-4 increased activity of the M2 macrophage marker, arginase-1. Moreover, BIO treatment increased Arg activity in the presence of LPS. n = 4, *p < 0.05, *t*-test.

Resident fibroblasts are known regulators of monocyte differentiation into macrophages. In co-cultures of THP-1 human monocytes and rat cardiac fibroblasts, increased monocyte adherence was observed, indicating macrophage differentiation (Fig. 4A,B). IL-10 is a M2 macrophages marker^16^, and co-cultured monocytes treated with BIO increased IL-10 expression (Fig. 4C). The increase in IL-10 expression was similar to co-cultured monocytes treated with IL-4, which is known to induce differentiation into M2 macrophages (Fig. 4C). In contrast, LiCl did not increase IL-10 expression. Treatment with BIO in the presence of LPS still produced an increase in IL-10 expression (Fig. 4D). Treatment of co-cultured monocytes with BIO or IL-4 was shown to increase Arg activity, which is a marker of M2 polarization (Fig. 4E). Treatment with LiCl did not increase Arg activity. In addition, treatment with BIO and LPS still produced an increase in Arg activity.

To verify that the effects of BIO are relevant *in vivo*, an *ex vivo* co-culture system was established using mouse bone marrow-derived monocytes (BMDMs) (Fig. 4F). BMDMs were cultured with conditioned media from rat cardiac fibroblasts, which induced macrophage differentiation (Fig. 4G). Treatment with BIO or IL-4 increased the expression of IL-10 (Fig. 4H). LPS produced an increase in IL-10 expression compared to BMDMs, but this increase was significantly less than observed with BIO treatment. LiCl did not increase IL-10 expression. BIO also increased IL-10 expression in the presence of LPS, unlike treatment with LPS and LiCl (Fig. 4I). As a positive control for M2 polarization, BMDMs were treated with a combination of TGF-β and IL-4. Treatment of differentiating BMDMs with BIO or IL-4, but not LiCl, increased Arg activity (Fig. 4J). Moreover, BIO increased Arg expression in differentiating BMDMs, even in the presence of LPS (Fig. 4J).

### BIO Rescues Cardiomyocyte Loss in Zebrafish Heart Failure

**Figure 5:**
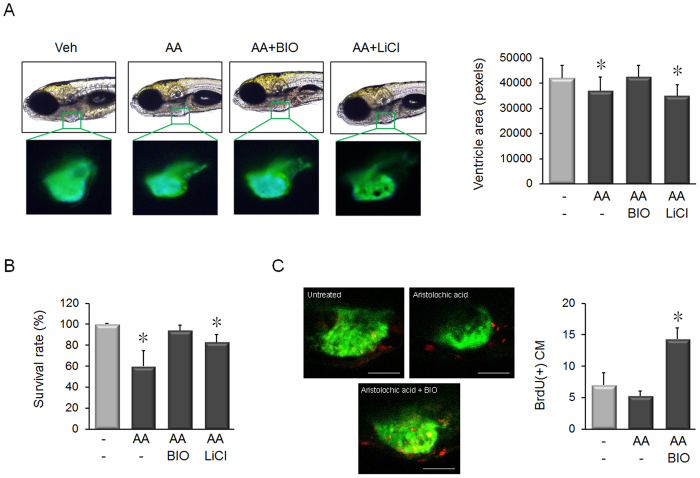
BIO rescues an animal model of experimental heart failure by inducing cardiomyocyte proliferation. n = 20/group. (**A**) Treatment of 72 hpf *Tg*(*cmlc2:GFP*) transgenic zebrafish larvae with 2.5 μM aristolochic acid (AA) for 3 hours induces cardiomyocyte death, which can be visualized as reduced heart size in treated larvae at 168 hpf. Treatment with 5 μM BIO, but not 20 mM LiCl increased ventricular size. *P < 0.05, *t*-test. (**B**) BIO treatment completely recovered survival rate in AA-treated animals. LiCl treatment increased survival rate, but not to the same degree as BIO treated animals. *p < 0.05, *t*-test. (**C**) Micrographs of the cardiac drug-treated larvae showed that BIO increased the number of proliferating cardiomyocytes in AA-treated animals, as detected by counting BrdU-immunostained cells in the GFP-positive cardiac region (cardiomyocytes = green; BrdU positive cells = red). *p < 0.05, *t*-test. Scale bar = 100 μm.

To investigate whether BIO produces therapeutic effects after cardiac injury *in vivo*, an optically transparent zebrafish model of heart failure was employed^17^. Zebrafish provide an ideal vertebrate system for initial testing of candidate drugs^18^,^19^. *Tg*(*cmlc2:GFP*) transgenic zebrafish showed reduced ventricular size after treatment with the cardiac toxin, aristolochic acid (AA) (Fig. 5A). Treatment with BIO, but not LiCl, recovered ventricular size and completely recovered survival rate (Fig. 5B). To test if BIO can induce cardiomyocyte proliferation, *Tg*(*cmlc2:GFP*) transgenic zebrafish were stained for proliferating cells using BrdU. A higher number of BrdU positive cells were present in the cardiac region of BIO-treated fish (Fig. 5C).

### BIO Promotes Favorable Cardiac Remodeling in Rat MI and Increases M2 Macrophages in the Infarction Zone

**Figure 6:**
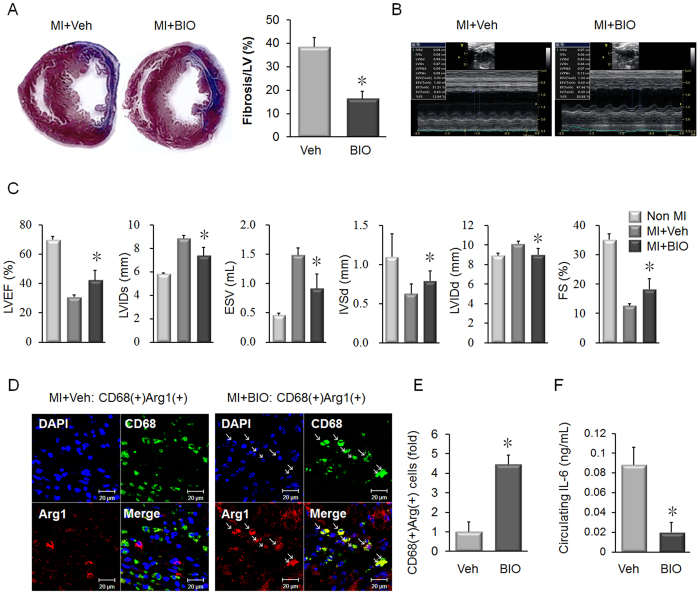
BIO improves cardiac remodeling with increased anti-inflammatory macrophages. (**A**) Masson’s trichrome staining of rat hearts two weeks after MI shows that 0.2 mg/kg BIO treatment reduced fibrosis and scar formation (blue staining). Quantification of fibrosis indicates that BIO treatment reduces scarring after MI. n = 4 in Non MI, n = 5 in MI + Veh, n = 8 in MI + BIO group, *p < 0.05, *t*-test. (**B**) Representative electrocardiography of the treated rats two weeks after MI. Treatment with BIO improved contractility after MI. (**C**) The cardiac function related parameters were improved in the BIO group two weeks after MI. N = 4 in Non MI, n = 5 in MI + Veh, n = 8 in MI + BIO group, *p < 0.05, *t*-test. IVSd, intraventricular septal width in diastole; IVSs, intraventricular septal width in systole; LVIDd, left ventricular internal dimension in diastole; LVIDs, left ventricular internal dimension in systole; LVPWd, left ventricular posterior wall thickness in diastole; LVPWs, left ventricular posterior wall thickness in systole; EDV, end-diastolic volume; ESV, end-systolic volume; EF, ejection fraction; SV, stroke volume; FS, fractional shortening. (**D**) Representative images of immunofluorescent staining for the macrophage marker CD68 (green) and Arg (red) in the infarct myocardium at 14 days post-MI. Arg-expressing M2 anti-inflammatory macrophages can be identified as yellow cells in the merged images. The BIO group was observed to contain greater numbers of M2 macrophages. (**E**) Quantification of anti-inflammatory CD68/Arg expressing macrophages showed that BIO group contained significantly higher numbers of M2 macrophages in the infarction zone. n = 3 in Non MI, n = 3 in MI + Veh, n = 6 in MI + BIO group, *p < 0.05, *t*-test. (**F**) Quantification of the pro-cardiac fibrosis cytokine, IL-6 in the sera of rats 2 weeks after MI. BIO treated rats showed decreased IL-6 levels. n = 5, *p < 0.05, *t*-test.

To test the effect of BIO on cardiac injury in mammals, a rat MI model was used. Male rats were treated with 0.2 mg/kg BIO or vehicle every day for 2 weeks after MI. BIO treated rats showed reduced ventricular fibrosis (Fig. 6A). Moreover, echocardiography analysis showed that BIO treatment significantly improved cardiac recovery and the indices of cardiac function at 2 weeks post-MI (Fig. 6B,C, Online Table 2). Left ventricular ejection fraction (EF %) was considerably improved (30.67% ± 1.659 in the Veh group vs. 41.31 ± 6.823 in the BIO group, *p* < 0.05), and fractional shortening (FS %) was also notably increased (12.62% ± 0.755 in the Veh group vs. 18.25 ± 3.525 in the BIO group, *p* < 0.05) by BIO treatment. Measurement of organ and body weight indicated that BIO produced no toxic effects in the rats (Online Table 3).

Our results indicated that BIO increased the expression of M2 macrophage markers (Fig. 4). Higher numbers of M2 macrophages were observed in the infarction zone of the BIO-treated group, as detected using the M2 marker, Arg (Fig. 6D,E). IL-6 is linked to the development of cardiac fibrosis and serum levels negatively correlate with prognosis in heart failure^20^. Using ELISA, it was observed that BIO treatment reduced serum IL-6 after MI (Fig. 6F).

## Discussion

Drug candidates that improve cardiac remodeling would possess significant therapeutic potential. The natural compound derivative, BIO, has been used as a GSK-3β inhibitor/Wnt pathway activator for numerous cardiac research applications, such as inducing cardiomyocyte differentiation in cardiac stem/precursor cells^21^. In this study, we establish BIO as a new drug that modulates the cardiac microenvironment to reduce scarring and improve cardiac performance after MI. Moreover, novel mechanisms of action for BIO were identified that are not observed for other GSK-3β inhibitors, such as LiCl. These novel mechanisms of BIO are directly related to the therapeutic effects observed in animal models.

Our results show that BIO induces proliferation in cardiomyocytes and inhibits proliferation in cardiac fibroblasts (Fig. 1). This important effect of BIO is emphasized by a previous study, which showed that adult cardiac fibroblasts induce only hypertrophy, not proliferation, in co-cultured cardiomyocytes^22^. The previous report by Tseng *et al*., showed that BIO increased cardiomyocyte proliferation by downregulating p27^5^. Our data showed that BIO increased p27 protein expression in fibroblasts, which explains the different effects of BIO in cardiomyocytes and fibroblasts. Immunoblotting showed that BIO decreased p21 protein expression in cardiac fibroblasts (Fig. 2E). These results indicate that p27 up-regulation after BIO treatment in cardiac fibroblasts overrides p21 to block proliferation. BIO treatment also reduced Akt activation (Fig. 2E). Akt activation induces cell proliferation and it has been shown that BIO treatment activates Akt in mesangial cells^23^. Thus, BIO differentially affects proliferation in various cell types.

In addition to inhibiting proliferation in cardiac fibroblasts, BIO also reduces motility (Fig. 2Ai). Interestingly, the known GSK-3β inhibitor, LiCl, did not reduce motility (Fig. 2Aii), indicating that BIO has significant additional effects in cardiac fibroblasts that are not related to GSK-3β inhibition. The role of GSK-3β inhibition in fibroblasts is controversial; increased fibrosis has been reported in numerous disease contexts, such as dermal and venous wall fibrosis^24^,^25^ and deletion of GSK-3β in cardiac fibroblasts increases scarring in the ischemic heart^26^. However, GSK-3β inhibition by over-expression of a dominant-negative isoform was protective in a murine model of heart failure^27^. Our data shows that BIO induces anti-fibrotic effects in cardiac fibroblasts, via reduced proliferation, inhibition of motility, decreased expression of the pro-fibrotic factors, CCL11 and CTFG, and increased expression of the anti-fibrotic factor, IL-10. Additionally, BIO also produced a small but significant reduction in the expression of TGF-β, which is considered a ‘master regulator’ of the fibrosis program (Fig. 2)^28^. Although our results show positive effects of BIO on modulating profibrotic factors, the signaling pathways that regulate fibrosis and myofibroblast differentiation are highly complex. Therefore, to further clarify the potential anti-fibrotic effects of BIO, additional analysis of key regulators is required, such as endothelin-1 and scleraxix^29^,^30^,^31^.

Resident macrophages are a component of the cardiac microenvironment and regulators of the remodeling process after MI^11^. M1 polarized macrophages clear wound debris and regulate the inflammatory response, whereas M2 polarized macrophages promote wound resolution and benefit cardiac remodeling when present in greater numbers^14^. Our results show that BIO reduced expression of the M1 marker, iNOS, in macrophages (Fig. 3). Moreover, the known GSK-3β inhibitor, LiCl, could not reduce iNOS expression to the same degree as BIO (Fig. 3B). Of note, it has previously been shown that increased iNOS expression after MI is linked to cardiac dysfunction and increased mortality^32^. In addition, treatment with iNOS inhibitors after experimental MI can reduce infarct size^33^.

During wound healing and remodeling post-MI, circulating monocytes ‘home’ to the infarction site and differentiate into macrophages. In other disease states, such as cancer, the role of microenvironment fibroblasts in modulating macrophage phenotype has been widely studied^34^. However, these cellular interactions are less studied in the cardiac microenvironment. Using a co-culture system, our results show that cardiac fibroblasts can induce monocyte differentiation into macrophages (Fig. 4B). To our knowledge, this is the first demonstration that cardiac fibroblasts secrete factors that induce monocyte differentiation. This ‘molecular crosstalk’ between different cell types is a major regulator of disease progression in cancer^35^ and our results show that crosstalk between cardiac fibroblasts and monocytes regulates macrophage differentiation. BIO treatment in our co-culture system increased expression of the M2 macrophage markers, IL-10 and Arg1 (Fig. 4C–E). IL-10 is a key anti-inflammatory cytokine and positively regulates cell-based repair after MI^36^. Moreover, the ratio of IL-10 expression compared to pro-inflammatory IL-6 is linked to the development of MI in patients^37^. To our knowledge, there is only one previous report investigating the interactions between cardiac fibroblasts and macrophages in co-culture, in which it was shown that murine macrophages stimulate pro-inflammatory IL-6 production and pro-fibrotic signaling in cardiac fibroblasts^38^. Our results show that BIO modulates the cellular ‘crosstalk’ between cardiac fibroblasts and differentiating macrophages to dramatically increase expression of IL-10 at a similar degree to the major M2 macrophage inducer, IL-4 (Fig. 4C,D). IL-10 is known to suppress the expression of iNOS in macrophages and induce expression of the M2 macrophage marker, Arg1. Our results show that BIO treatment also induced Arg1 expression in differentiating bone-marrow derived and human macrophages (Fig. 4E,J). Arg1-expressing macrophages are known to inhibit liver fibrosis in mice.

The zebrafish model has proved an invaluable resource for the primary validation of new bioactive compounds *in vivo*. The optically transparent zebrafish heart failure model used in our study demonstrated that BIO induces cardiomyocyte proliferation to effectively compensate for toxicity induced by AA. In contrast, the GSK-3β inhibitor LiCl was not as effective as BIO at rescuing the zebrafish. The zebrafish data represents the first demonstration that BIO induces cardiomyocyte proliferation *in vivo* (Fig. 5C). Moreover, the lack of toxicity in BIO-treated zebrafish supports the further drug development of BIO, because mammals and zebrafish show strong similarities in their response to toxic agents^39^.

Our zebrafish-based data validated the testing of BIO in a mammalian model of MI. BIO treatment improved cardiac remodeling and reduced fibrotic scar formation (Fig. 6). The improvement in cardiac fibrosis produced by BIO compared favorably with other experimental interventions that enhance remodeling. For example, mesenchymal-derived cell (MSC) therapy for MI produced an average reduction of 6.67% in fibrosis level^40^, compared to 11.72% for BIO treated rats. Increasing the numbers of M2 macrophages is known to improve cardiac remodeling and recovery after MI^14^. In the context of cardiac fibrosis, IL-6 acts as a pro-inflammatory cytokine that is both produced by M1 macrophages and cardiac fibroblasts to induce M1 polarization^41^. BIO treatment reduced serum levels of pro-inflammatory IL-6 (Fig. 6F), which provides a potential mechanism to explain the increased numbers of M2 macrophages in the infarction zone.

BIO was effective in the rat and zebrafish models without producing noticeable toxicity. However, to translate the BIO compound into clinical applications, its potential toxicity in different organs should be investigated more rigorously in animal models. It should be noted that many patients have received long-term treatment with the GSK-3β inhibitor, lithium, without producing significant effects on the cardiovascular system. However, recent studies have indicated that GSK-3 isoform selective (α- or β-) small molecule inhibitors should be developed to prevent myocardial fibrotic remodeling^42^.

Our study indicates that BIO has potential to be developed as a therapy for improving remodeling post-MI, although the effects are only partial. There are currently few drug options available for patients, despite numerous lead compounds showing promise in animal models. Angiotensin-converting enzyme (ACE) inhibitors, aldosterone inhibitors and the beta blocker, carvedilol, have shown effectiveness in MI patients, but may not actually reverse the remodeling process^43^,^44^. Therefore, although BIO has shown promising results in this study, it is still at an early stage of development as a therapeutic for attenuating remodeling post-MI.

Our study shows that BIO can increase proliferation in zebrafish and rat cardiomyocytes. The ability for cardiomyocytes to re-enter the cell cycle varies amongst species and is very limited in humans^45^. For example, zebrafish cardiomyocytes analyzed in our study readily re-enter the cell cycle after heart trauma. Moreover, rat neonatal cardiomyocytes used for our mammalian assays of BIO treatment show greater proliferative potential than adult cardiomyocytes^5^. Therefore, further study of the effects of BIO on cardiomyocyte proliferation in higher mammalian species is warranted to assess whether this compound has the potential to be effective in human cardiomyocytes.

A recent study demonstrated that activation of Notch 1 by its ligand, Jagged1, produced distinct responses that are dependent on cell types in the stressed adult mouse heart^46^. Activated Notch1 in Jagged1 transgenic mice reduced both hypertrophy and fibrosis in response to pressure overload. Interestingly, upregulated Notch1 increased the number of stem cell antigen-1-posivite cardiac precursor cells, and decreased the number of myofibroblasts in the stressed heart. In the Notch1 activated heart, pressure overload-induced Akt phosphorylation was reduced, which could explain the decrease in cardiac hypertrophy. Further identification of the relationship between BIO and the Notch1 signaling pathway in cardiomyocytes and cardiac fibroblasts may further clarify mechanism of action of BIO in the damaged cardiac microenvironment.

Adult cardiac precursor cells (CPCs) have been identified as an important cell population that contributes to repair after damage to the myocardium^47^. The Wnt signaling pathway has been shown to be regulate the contribution of CPCs to cardiac repair^48^. Wnt pathway inhibition after chronic left anterior descending coronary artery ligation reduced mortality and improved remodeling. However, BIO activates the Wnt signaling pathway via the inhibition of GSK-3β. To our knowledge, there is no published data concerning the effects of BIO on adult CPC renewal or differentiation into cardiomyocytes. In light of our results showing the beneficial effect of BIO on remodeling post-MI, an investigation of how BIO modulates CPC phenotype would be an interesting area for further research.

**Figure 7:**
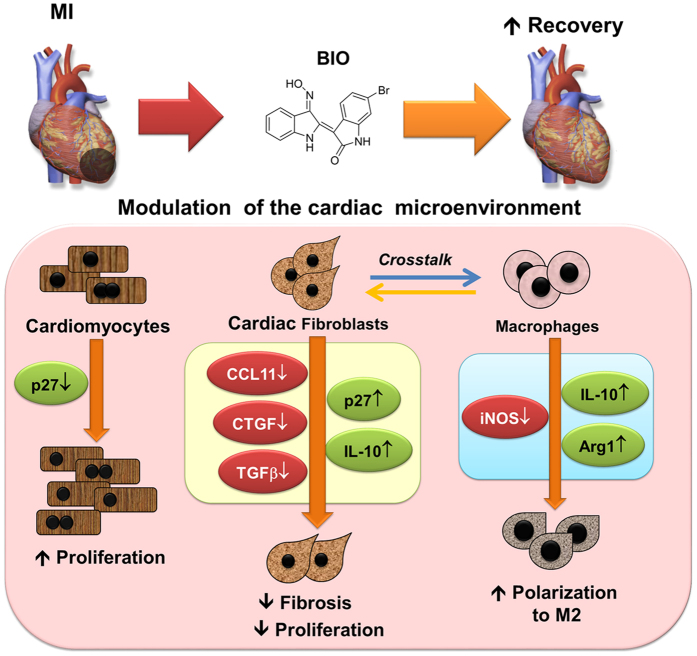
BIO modulates multiple aspects of the cardiac microenvironment to improve remodeling after MI. Schematic of the pleiotropic effects of BIO on cardiac cells to improve remodeling after MI. BIO induces proliferation in refractory cardiomyocytes, providing a therapeutic opportunity to replace a portion of the dead cardiomyocytes. Conversely, in cardiac fibroblasts BIO up-regulates p27 and reduces proliferation. BIO also inhibits the pro-fibrotic factors CCL11, CTGF and TGF-β, while upregulating the anti-fibrotic factor, IL-10. BIO also modulates the cellular crosstalk between cardiac fibroblasts and differentiating monocytes, via upregulation of IL-10 and Arg1, and downregulates the pro-inflammatory iNOS. Overall, in the cardiac microenvironment BIO modulates cell proliferation, fibrosis and monocyte differentiation to improve recovery after MI. The image of the heart (https://commons.wikimedia.org/wiki/File:Blalock_Taussig_Shunt_-_Subclavian_to_Pulmonary.png) is obtained from Wikipedia and used under the Creative Commons Attribution-Share Alike 4.0 International license (http://creativecommons.org/licenses/by-sa/4.0/).

In summary, this study has shown that BIO induces multiple, beneficial effects on the major cell types comprising the cardiac microenvironment to reduce fibrosis and improve remodeling after MI. Many of these effects were not observed after treatment with the commonly used GSK-3β inhibitor, LiCl, confirming that BIO produces pleiotropic effects in these cells types that are additional to its reported activity against GSK-3β. We show that BIO treatment enhances the proliferation of cardiomyocytes and reduces the proliferation of cardiac fibroblasts via differential regulation of p27 expression. BIO treatment produces anti-fibrotic effects in cardiac fibroblasts, such as reduced motility, down-regulation of CCL11 and up-regulation of IL-10. In monocytes, BIO treatment promotes monocyte differentiation into anti-inflammatory M2 macrophages by strongly inducing IL-10 expression in cardiac fibroblast co-cultures. These beneficial effects of BIO can be observed in a zebrafish heart failure model and a rat MI model. The pleiotropic effects of BIO in the cardiac microenvironment are summarized in Fig. 7. To our knowledge, no previously reported compound has been shown to produce this combination of differential, positive effects on the cardiac microenvironment. Thus, our study represents a significant reappraisal of the biological activity and therapeutic potential of BIO. With ischemic heart disease remaining a leading case of mortality/morbidity and health care expense, this study supports the further development of BIO as a drug to target the cardiac microenvironment and improve remodeling after MI.

## Materials and Methods

All experiments conformed the NIH Guide for the Care and Use of Laboratory Animals published by the US National Institute of Health (NIH publication, 8th edition, 2011) and were approved by the Chonnam National University Institutional Animal Care and Use Committee (study approval code: CNU IACUC-H-2014-23). A description of the following methods are available in the Online Supplement: Antibodies and reagents, cell lines, isolation and culture of neonatal left ventricular cardiomyocytes and cardiac fibroblasts, measurement of cell proliferation, cardiomyocyte: cardiac fibroblast co-culture system, immunocytochemistry, monolayer scratch assay, RT-PCR, quantitative real-time PCR, isolation and culture of primary bone marrow-derived monocytes, cardiac fibroblast and monocyte co-culture, arginase assay, zebrafish model of heart failure, rat model of acute MI, histological staining of fibrosis, echocardiography of ventricular function, immunohistochemistry of cardiac tissue, interleukin-6 enzyme-linked immunosorbent assay, statistical analysis.

## Additional Information

**How to cite this article**: Kim, Y. S. *et al*. Natural product derivative BIO promotes recovery after myocardial infarction via unique modulation of the cardiac microenvironment. *Sci. Rep.* **6**, 30726; doi: 10.1038/srep30726 (2016).

## Supplementary Material

Supplementary Information
