# Supplementary material for: Natural product derivative BIO promotes recovery after myocardial infarction via unique modulation of the cardiac microenvironment
Source: Sci Rep. 2016 Aug 11;6:30726. doi: 10.1038/srep30726 (PMC4980696; doi:10.1038/srep30726)
Supplement: Supplementary Information [file srep30726-s1.pdf]

Natural product derivative BIO promotes recovery after myocardial infarction via unique modulation of the cardiac microenvironment

Yong Sook Kim[#1], Hye-yun Jeong[#2], Ah Ra Kim[#3], Woong-Hee Kim[3], Haaglim Cho[3], JungIn Um[3], Youngha Seo[3], Wan Seok Kang[2], Suk-Won Jin[3], Min Chul Kim[4], Yong-Chul Kim[3], Da-Woon Jung*[3], Darren R. Williams*[3], Youngkeun Ahn*[2,4]

[1]Biomedical Research Institute, [2]Research Laboratory of Cardiovascular Regeneration, Chonnam National University Hospital, Gwangju, 501-757, Republic of Korea

[3]School of Life Sciences, Gwangju Institute of Science and Technology, 1 Oryong-Dong, Buk-Gu, Gwangju 61005, Republic of Korea

[4]Department of Cardiology, Chonnam National University Hospital, Gwangju, Korea

To whom correspondence should be addressed: 1) Da-Woon Jung, School of Life Sciences, Gwangju Institute of Science and Technology, 1 Oryong-Dong, Buk-Gu, Gwangju 61005, Republic of Korea. Tel: +82-62-715-3554; email: jung@gist.ac.kr 2) Darren R. Williams, School of Life Sciences, Gwangju Institute of Science and Technology, 1 Oryong-Dong, Buk-Gu, Gwangju 61005, Republic of Korea. Tel: +82-62-715-2509; email: darren@gist.ac.kr 3) Youngkeun Ahn, Chonnam National University Hospital, Gwangju, 61469, Republic of Korea. email: cecilyk@jnu.ac.kr

[#]These authors contributed equally to this study.

SUPPLEMENTAL INFORMATION

Materials and Methods

Antibodies and reagents

AntiotensinII (AngII), aristolochic acid (AA), bromodeoxyuridine (BrdU), (2'Z,3'E)-6-Bromoindirubin-3′-oxime (BIO), lipopolysaccharide (LPS), lithium chloride and methylthiazolyldiphenyl-tetrazolium bromide (MTT) and dimethyl sulfoxide (DMSO) were purchased from Sigma, USA. Human and mouse interleukin 4 (IL-4) was purchased from ProSpec, USA. Transforming growth factor-β (TGF-β) was purchased from R&D systems, USA. The following antibodies were used in this study: anti-Akt (Cell Signaling Technology, USA), anti-phosphorylated Akt (Cell Signaling Technology, USA), anti-Bcl2 (Santa Cruz, USA), anti-GAPDH (Santa Cruz, USA), anti-iNOS (Cell Signaling Technology, USA), anti-p21 (Santa Cruz, USA), anti-p27 (Santa Cruz, USA), arginase-1 (LifeSpan BioScience, USA), CD206 (Abcam, USA), CD68 (Biomedicals, Switzerland), and phosphorylated histone H3 (Abcam, USA).

Cell lines

RAW264.7 murine macrophages cell were purchased from the Korean Cell Bank (Seoul, Korea). Cells were cultured in DMEM media supplemented with 10% FBS, without antibiotics. The human THP-1 monocyte cell line was cultured in RPMI 1640 media supplemented with 10% FBS, 1% PenStrep and 50 mM β-mercaptoethanol. THP-1 monocytes were differentiated into macrophages by 72 h treatment with 200 nM phorbol myristate acetate (PMA) without β-mercaptoethanol.

Isolation and culture of neonatal left ventricular cardiomyocytes and cardiac fibroblasts

Primary cardiomyocytes and cardiac fibroblasts were isolated from 2 days-old Sprague-Dawley rats. Briefly, neonatal ventricles from neonatal rats euthanized by decapitation were separated and washed in cold-PBS, chopped using a scalpel, and digested with 0.1% collagenase type 2 (210 U/mL, Sigma-Aldrich, USA) and pancreatin (0.6 mg/mL, Gibco, USA) for 30 minutes with mild stirring. The supernatant was collected for centrifugation through a Percoll gradient (Sigma-Aldrich, USA) at 1000 rpm for 5 min. The cardiac cell layer was collected and cultured in a flask with Dulbecco's Modified Eagle's Medium (DMEM; Invitrogen, USA) supplemented with 10% heat-inactivated fetal bovine serum (FBS). After 1 hour, the non-adherent cardiomyocyte population was removed from the adherent cardiac fibroblasts. The proliferation culture for cardiac fibroblasts was DMEM supplemented with 10% FBS and 1% PenStrep (50 units/mL penicillin and 50 µg/mL streptomycin). The fibroblasts were used for experiments at passage 2 or passage 3.

Cell proliferation assay using BrdU labelling

Cardiomyocytes and cardiac fibroblasts were treated with BIO in the presence of BrdU (10 µM). After 2 or 5 days, cells were fixed with 4% para-formaldehyde or ice-cold methanol for 10 minutes and were washed with PBS three times. After permeabilization by 0.1% Triton X-100 for 10 minutes and blocking with 5% BSA for 1 hour, primary antibodies were incubated overnight at 4 ℃ followed by sequential incubation with secondary antibody conjugated with Alexa-fluor-488 or Alexa-fluor-594 (Molecular Probe, USA, 1:300). Primary antibodies used for immunofluorescence included cardiac troponin I (Santa Cruz, USA, 1:100) and BrdU (Santa Cruz, USA, 1:100). After washing with PBS, the slides were mounted with medium containing DAPI (Invitrogen, USA) and were observed under fluorescence microscopy. For quantification of the proliferating cells, BrdU(+) cells were counted. To distinguish the cell types, BrdU(+)cTnI(+)DAPI(+) cells were regarded as proliferating cardiomyocytes and BrdU(+)cTnI(-)DAPI(+) cells were regarded as proliferating cardiac fibroblasts.

MTT assay for cell proliferation

Cell proliferation was assessed using MTT assay. Briefly, 0.1 volume of MTT (1 mg/mL) was added to cell culture medium and incubated for another 4 hrs. The medium was removed and 100 μL of DMSO was added into each well, and the plate was gently rotated to completely dissolve the precipitation. The absorbance was measured at 540 nm.

Cardiomyocyte: cardiac fibroblast co-culture system

Primary cardiomyocytes and cardiac fibroblasts were purified and cultured as described above. Cells were counted and mixed before seeding at a ratio of 1:1 (by cell number). Cells were seeded on sterile glass cover slips (100 mm diameter) in 12-well plates. The seeding density was 30,000 cells/well in 1 mL (for 2 days treatment) and 20,000 cells/well in 1 mL (for 5 day treatment).

Immunofluorescence staining

After treatment of RAW264.7 cells or cardiac fibroblasts, cells were fixed with 4% para-formaldehyde or ice-cold methanol for 10 minutes and washed with PBS three times. After permeabilization by 0.1% Triton X-100 for 10 minutes and blocking with 5% BSA for 1 hour, primary antibodies were incubated overnight at 4 ℃ followed by sequential incubation with secondary antibody conjugated with Alexa-fluor-488 or Alexa-fluor-594 (Molecular Probe, USA, 1:200). After washing with PBS, the slides were mounted with medium containing DAPI (Invitrogen, USA) and observed under fluorescence microscopy.

Monolayer scratch assay

The scratch assay was carried out as previously described.[1] Briefly, cardiac fibroblasts were seeded onto six-well plates and allowed to adhere overnight (achieving >90% confluence). The monolayer was wounded with a 200 μL plastic pipette tip. Cells were washed once, and fresh culture medium was added with or without the compound of interest. The cells were allowed to invade the wound and counted by light microscopy 12 hr later. The rate of migration into the scratch was quantified by measuring the width of the wound at five different sites every 24 hr using light microscopy (CKX41, Olympus) and iSolution Lite 9.1 image capture software.

RT-PCR

RNA was harvested from cells using the TRI reagent, following the manufacturer's instructions (Sigma, MO, USA). 0.5 μg RNA and 100 pmole oligo dT_{16} was used for reverse transcription (AccuPower® RT PreMix; Bioneer). 2.5 μL RT product was used for PCR (AccuPower® PCR PreMix; Bioneer). Densitometric analysis was performed using the Scion Image program (Scion Corporation, USA). The PCR primers used in this study are shown in Table 1.

Quantitative PCR

The transcript level of cytokines was analyzed by quantitative real-time RT-PCR using StepOnePlus Real Time PCR System (Applied Biosystems, UK). Cells were washed twice in PBS and were frozen at −70 ºC until the RNA was isolated. Total RNA was isolated using Trizol according to the manufacturer's protocol and was reverse transcribed to prepare cDNA using AccuPower® RT PreMix (Bioneer Corporation). The cDNA obtained was subjected to real-time PCR according to the manufacturer's instructions with the following modifications. PCR was performed in triplicate in a total

volume of 20 μL of 2X Power SYBR® Green PCR Master Mix(Applied Biosystems, UK) containing each 200 nM (final concentration) of specific primer and 1 μL of cDNA. PCR amplification was preceded by incubation of the mixture for 10 min at 95 ℃ and the amplification step consisted of 40 cycles of denaturation, annealing and extension. The denaturation was performed for 15 s at 95 ℃, annealing was done for 1 m at 60 ℃ and the extension was performed at 72 ℃ for 20 s with fluorescence detection at 72 ℃ after each cycle. After the final cycle, melting-point analysis of all of the samples was performed within the range of 60–95 ℃ with continuous fluorescence detection. A specific cDNA sample was included in each run and served as a reference for the comparison between runs. The expression level of GAPDH was used for normalization while calculating the expression levels of all of the other genes. Results were expressed as the relative expression level for each gene. The synthesized cDNAs were amplified by quantitative real time PCR (qRT-PCR) or standard PCR.

Western blotting

Whole cell lysates were harvested using lysis buffer (20mM Tris-HCl pH 7.4, 0.1mM EDTA, 150mM NaCl, 1mM phenylmethylsulfonyl fluoride (PMSF) and 1mg/ml leupeptin) on a rotation wheel for 1 h at 4℃. After centrifugation at 10,000*g* for 10min, the supernatant was prepared as a protein extract. Equal concentrations of proteins were fractionated by electrophoresis on 8% -12% acrylamide gels and were transferred onto a polyvinylidene fluoride membrane (Merck Millipore, Darmstadt, Germany) membrane, followed by blotting with antibodies followed by secondary staining with horseradish peroxidase-conjugated immunoglobulin G. Protein expression was detected using an Image Reader (LAS-3000 Imaging System, Fuji Photo Film, Tokyo, Japan). The expression level was quantified by ensitometric analysis using Scion Image program.

Isolation and culture of primary bone marrow-derived monocytes

Two young adult (6 weeks) B6 female mice were sacrificed by cervical dislocation and the femur and tibia bones were dissected. The ends of the bones were cut and the marrow was washed out with RPMI 1640 media using a 1 mL insulin syringe (26 gauge needle). Marrow cells were transferred to a 50 mL sterile tube and sieved through a 70 µm mesh to remove debris. 10 mL red blood cell lysis buffer (BioLegend, USA) was, followed by incubation on ice for 5 min. Bone marrow cells were harvested by centrifugation at 1200 rpm for 10 min. The supernatant was removed and cells were washed two, times with 10 mL PBS. Cells were counted and seeded in 6-well culture plates at a density of 10^6 cells/well. Cells were cultured with RPMI 1640 media supplemented with 10% FBS, 1% P/S, 2 mM L-glutamine, 1X non-essential amino acids, 1M HEPES and 50 µM β-mercaptoethanol. 2 h later, non-adherent cells were transferred to a 6-well ultra-low attachment surfaced plate (Corning incorporated, USA) and stimulated with 20 ng/mL murine macrophage colony stimulating factor (M-CSF) for 4 days, with one media change on the 3rd day of treatment. The non-adherent cells were harvested and monocytes purified using CD117-coated magnetic beads (Miltenyi Biotec, UK), which remove non-monocytes from the cell population.

Cardiac fibroblast and monocyte co-culture

Rat cardiac fibroblasts were derived as described above. The human THP-1 monocyte cell line was seeded in the lower chamber of a 6-well plate transwell system (0.4 µm, Corning) at a density of $2x10^5$ cells/well. Cardiac fibroblasts were seeded in the upper chamber at a density of 10^5 cells/well. Cells were cultured in 3 mL media per well consisting of THP-1 media and cardiac fibroblast media at a 2:1 ratio.

Arginase assay

The assay was carried out in accordance with the manufacturer's instructions (BioVision Inc., USA). Adherent macrophages were harvested from the co-culture system by cell scraping. 0.25 ug/mL protein lysate was used for the assay.

Zebrafish model of heart failure

The zebrafish model was based on a previous study.[2] Briefly, embryos from mated *Tg(cmlc2:GFP)* transgenic zebrafish were grown in distilled water supplemented with 0.06 g/L sea salt and staged by timing in hours post fertilization (hpf). Care and treatment of animals were conducted in accordance with guidelines established by the Animal Care and Ethics Committees of the Gwangju Institute of Science and Technology. At 72 hpf, heart formation is complete[3] and embryos were treated with the cardiomyocyte toxin, aristolochic acid[4], for 3 h. 5 μM BIO or 5 mM lithium chloride were added at the same time as aristolochic acid. The treatment concentration of lithium chloride was based on a previous study in zebrafish.[5] After treatment, drugs were removed by 5 washes with egg water. At 168 hpf, zebrafish were visually assessed for survival and cardiac morphology using microscopy (LEICA DMI3000 B).

For staining with bromodeoxyuridine, zebrafish were treated with 10 mM BrdU with 1% DMSO in 1X E3 buffer for 1 h at 28.5℃, using a fish incubator. The fish were euthanized and fixed in 4% paraformaldehyde for 2 h. Fish larvae were washed 3 times with PBDT buffer (0.1% Tween-20, 1% DMSO in PBS) and dehydrated in 100% methanol for 1 h at -20℃. The larvae were then rehydrated in a graded methanol series: 75%, 50%, 25% for 20 min each and wash in PBDT for 20 min. Larvae were then digested with proteinase K (10 μg/mL) for 20 min. After three washes with PBDT, larvae were re-fixed in 4% parformaldehyde for 20 min. Larvae were then washed with PBDT and incubated with 12. 2N HCl for 1 h at RT, followed by 3 washes with PBDT and incubation with 10% normal goat serum in PBDT for 1 h. Larvae were then incubated with mouse anti-BrdU IgG in blocking serum overnight at 4℃. Larvae

were washed three times with PBDT and incubated in goat anti-mouse IgG conjugated to Alexa-fluor 594 for 5 h in the dark. After washing with PBDT, stained larvae were mounted and imaged using confocal microscopy (Olympus FluoView^{TM} FV1000).

Rat model of acute MI

The study was reviewed and approved by the Chonnam National University Institutional Animal Care and Use Committee (study approval code: CNU IACUC-H-2014-23). Male 8-week old Sprague-Dawley rats (weight 200-230 g) were purchased from the company Jung Ang Animals (Seoul, Korea). MI was induced by permanent ligation of the left anterior descending coronary artery, as follows: rats were anesthetized with an intramuscular injection of ketamine (50 mg/kg), and xylazine (5 mg/kg), intubated, and mechanically ventilated. The proximal left anterior descending coronary artery was ligated. Finally, the heart was repositioned in the chest, and the chest was closed. The animals remained in a supervised setting until becoming fully conscious. After induction of MI and recovery from anesthesia, animals were administered with saline or BIO (0.2 mg/kg of body weight; dissolved in PBS + ethyl alcohol (1:1 vol/vol)) every 24 h via intraperitoneal injection for 2 weeks. Experimental groups were as follows: Non MI, MI + vehicle, and MI+BIO.

Echocardiography of ventricular function

After 2 weeks of treatment with BIO or saline, left ventricular function was assessed by echocardiography. Rats were anesthetized with an intramuscular injection of ketamine (50 mg/kg) and xylazine (5 mg/kg). Echocardiographic studies were performed with a 15-MHz linear array transducer system (iE33 system, Philips Medical Systems) by an expert who was unaware of the experimental conditions to exclude bias. Two-dimensional guided M-mode of the LV was obtained from the

parasternal view. LV cavity dimension was measured, and percentage change in LV dimension [fractional shortening (FS), LV%FS] was calculated as: LV%FS = [(LVEDD - LVESD)/LVEDE] × 100, where LVEDD is left ventricular end-diastolic diameter and LVESD is left ventricular end-systolic diameter. LV% ejection fraction (EF) was calculated as: LV%EF = [(EDV - ESV)/EDV] × 100, where EDV is LV volume at end-diastole and ESV is LV volume at end-systole. LV volume was estimated by the area-length method. After echocardiography, heart was quickly removed, fixed in formalin, and embedded in paraffin for further histological studies.

Histological staining of fibrosis

Cardiac fibrosis after acute MI was measured by Masson's Trichrome staining, and fibrotic areas were measured by visualizing blue-stained fibrosis deposits by using NIS-Elements Advanced Research program (Nikon, Japan). The percentage of ventricular fibrosis was calculated as the blue-stained area divided by left ventricular area.

Immunohistochemistry of cardiac tissue

For immunohistochemical analysis, slides were treated with 3% hydrogen peroxide in PBS for 10 minutes at room temperature to block endogenous peroxidase activity. After nonspecific binding was blocked with 5% normal goat serum (Sigma-Aldrich, USA), the slides were incubated with primary antibodies against CD68 (Biomedicals, Switzerland, 1:100) or Arg-1 (Abcam, USA, 1:100) for overnight. Sections were washed with PBS three times, and then incubated for 1 hour with Alexa-fluor 488 or 594 secondary antibodies. After washing, the slides were coverslipped with mounting medium (VectaMount mounting medium, Vector Labs Inc., USA). Immunofluorescence was detected by using a Carl-Zeiss confocal microscope. Images were obtained by using Zeiss LSM version 3.2 SP2 software.

Interleukin-6 enzyme-linked immunosorbent assay

Serum levels of interleukin-6 (IL-6) in rats subjected to MI was measured by ELISA (eBioscience, USA). Whole blood was collected from rats by cardiac puncture immediately after sacrifice (between 3 – 5 mL was collected) using e-tubes (Axygen, USA) and kept on ice. Blood was centrifuged at 3500 rpm for 15 min at 4 ℃. The serum was transferred to fresh e-tubes and stored at -80 ℃. 100 μL undiluted serum was used for ELISA.

Statistical analysis

All experiments were performed a minimum of three times. The data are presented as means ± standard deviation. Differences were analyzed by Student's *t*-test or one-way ANOVA, followed by Tukey post-hoc test. *P*-values less than 0.05 were considered significant. Analysis was performed using GraphPad Prism (version 5.03, GraphPad Software Inc.).

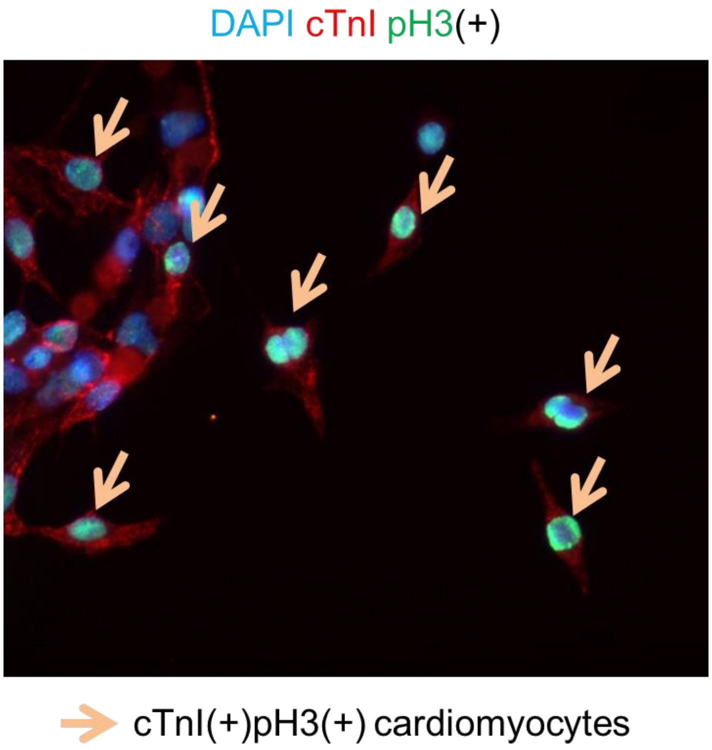

Supplementary Figure 1. Neonatal rat cardiomyocytes were treated with BIO for 2 days, and proliferating cardiomyocytes were detected with immunofluorescence staining with antibody against phosphorylated histone H3.

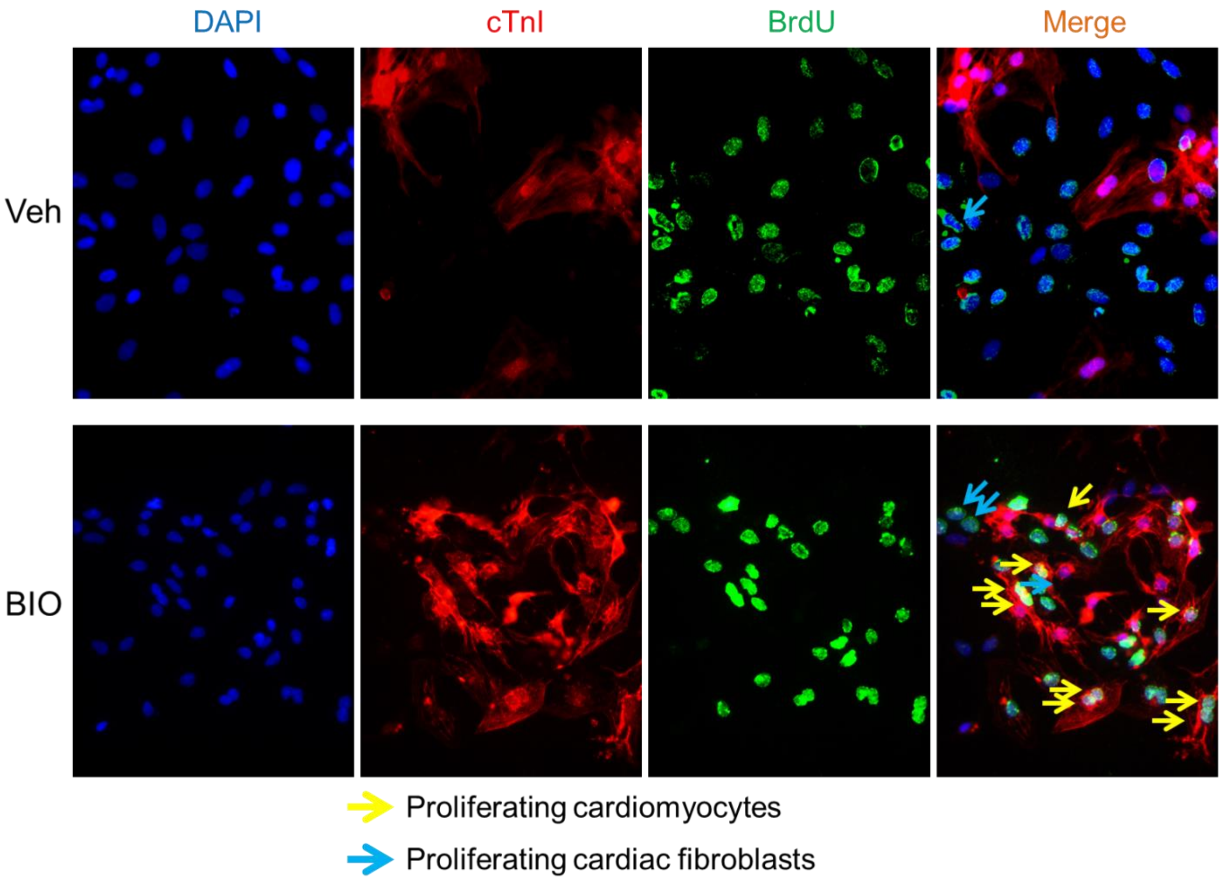

Supplementary Figure 2. Cocultures of neonatal rat cardiomyocytes and cardiac fibroblasts were treated with vehicle or 5 μM BIO for 5 days, and immunofluorescence staining with BrdU was performed to measure the proliferating cells. Cardiac troponin I (cTnI)-positive cell was regarded as a cardiomyocyte, and cTnI-negative cell was as a cardiac fibroblast, and BrdU-positive cell as a proliferating cell.

Table 1: PCR primers used in this study (in alphabetical order)

Gene	Species	Primer sequence or product code	Product size (bp)
CCL11	Rat	Forward 5' -GCG CTT CTA TTC CTG CTG CTC-3'<br>Reverse: 5'-GTG CTT TGT GGC ATC CTG GA-3'	245
CD68	Mouse	Forward 5'-TTC TGC TGT GGA AAT GCA AG-3'<br>Reverse: 5'-TCA CGG TTG CAA GAG AAA CA-3'	128
CTGF	Rat	Forward 5'-ACCGACCTCCTCCAGACGGC-3';<br>Reverse: 5'-CGTCCAGCACCAGGCTCACG-3'	317
GAPDH	Human	Forward 5'-ACAACTTTGGTATCGTGGAAGG-3'<br>Reverse: 5'-GCCATCACGCCACAGTTTC	101
GAPDH	Mouse	Forward 5'-TGA TGA CAT CAA GAA GGT GAA G-3'<br>Reverse 5'-TCC TTG GAG GCC ATG TAG GCC AT-3'	200
IL-10	Mouse	Forward 5'-ATA ACT GCA CCC ACT TCC CA-3'; Reverse: 5'-TCA TTT CCG ATA AGG CTT GG-3'	177
IL-10	Rat	Forward 5'-ATG CTC CTA GAG CTG CGG ACT G-3'<br>Reverse: 5'-CTC ACC CAG GGA ATT CAA ATG C-3'	225
IL-10	Human	Forward 5'-GGT TGC CAA GCC TTG TCT GA-3'<br>Reverse: 5'-AGG GAG TTC ACA TGC GCC T	101
p21	Mouse	Santa Cruz Biotechnology (sc-29428-PR)	524
p27	Mouse	Santa Cruz Biotechnology (sc-29430-PR)	437
TGF-β	Rat	Forward 5'-ATG GTG GAC CGC AAC AAC G-3';<br>Reverse: 5'-CAG CAG CCG GTT ACC AAG GT-3'	225

Table 2. Male Sprague-Dawley rats: IP BIO injection for 2 weeks post-MI

Rat ID	Basal BW (g)	BW at 2 weeks (g)	Heart (g)	Liver (g)	Lung (g)	Kidney (g)	Spleen (g)
PBS 1	275	345	1.07	11.73	1.40	3.00	0.94
PBS 2	280	360	1.18	13.44	1.65	2.95	1.11
PBS 3	260	350	1.06	13.70	1.42	2.53	1.07
Mean	271.67	351.67	1.10	12.96	1.49	2.83	1.04
SD	10.41	7.64	0.07	1.07	0.14	0.26	0.09
BIO 1	295	350	1.22	14.35	1.91	3.26	0.94
BIO 2	280	330	0.96	13.59	1.64	2.62	0.89
BIO 3	285	310	1.06	12.92	2.60	2.74	1.02
BIO 4	285	340	1.03	13.61	2.17	2.89	0.88
BIO 5	275	325	1.06	12.69	1.59	2.85	1.01
Mean	284.00	331.00	1.07	13.43	1.98	2.87	0.95
SD	7.42	15.17	0.10	0.65	0.42	0.24	0.07
p	0.095	0.075	0.577	0.456	0.102	0.810	0.140

Table 3. Echocardiographic analysis at 2 weeks post-MI

	Non-MI	MI + Vehicle	MI + BIO
IVSd (cm)	0.11 ± 0.029	0.06 ± 0.013	0.08 ± 0.013*
IVSs (cm)	0.14 ± 0.022	0.07 ± 0.014	0.08 ± 0.028
LVIDd (cm)	0.89 ± 0.022	1.01 ± 0.026	0.90 ± 0.066*
LVIDs (cm)	0.58 ± 0.012	0.89 ± 0.024	0.74 ± 0.074*
LVPWd (cm)	0.13 ± 0.014	0.08 ± 0.010	0.09 ± 0.023
LVPWs (cm)	0.17 ± 0.012	0.07 ± 0.005	0.12 ± 0.019*
EDV (mL)	1.52 ± 0.111	2.14 ± 0.153	1.56 ± 0.308*
ESV (mL)	0.46 ± 0.035	1.48 ± 0.121	0.91 ± 0.251*
EF (%)	69.85 ± 2.360	30.67 ± 1.659	41.31 ± 6.823*
SV (mL)	1.06 ± 0.102	0.65 ± 0.052	0.65 ± 0.116
FS (%)	35.18 ± 1.89	12.62 ± 0.755	18.26 ± 3.525*

*; $p < 0.05$ vs. MI+Vehicle group

IVSd, intraventricular septal width in diastole; IVSs, intraventricular septal width in systole; LVIDd, left ventricular internal dimension in diastole; LVIDs, left ventricular internal dimension in systole; LVPWd, left ventricular posterior wall thickness in diastole; LVPWs, left ventricular posterior wall thickness in systole; EDV, end-diastolic volume; ESV, end-systolic volume; EF, ejection fraction; SV, stroke volume; FS, fractional shortening

References for Supplementary Information

1. Jung DW, Kim WH, Seo S, Oh E, Yim SH, Ha HH, Chang YT and Williams DR. Chemical targeting of GAPDH moonlighting function in cancer cells reveals its role in tubulin regulation. *Chem Biol*. 2014;21:1533-45.
2. Lam NT, Currie PD, Lieschke GJ, Rosenthal NA and Kaye DM. Nerve growth factor stimulates cardiac regeneration via cardiomyocyte proliferation in experimental heart failure. *PLoS One*. 2012;7:e53210.
3. Carney SA, Chen J, Burns CG, Xiong KM, Peterson RE and Heideman W. Aryl hydrocarbon receptor activation produces heart-specific transcriptional and toxic responses in developing zebrafish. *Mol Pharmacol*. 2006;70:549-61.
4. Huang CC, Chen PC, Huang CW and Yu J. Aristolochic Acid induces heart failure in zebrafish embryos that is mediated by inflammation. *Toxicological sciences : an official journal of the Society of Toxicology*. 2007;100:486-94.
5. Nery LR, Eltz NS, Martins L, Guerim LD, Pereira TC, Bogo MR and Vianna MR. Sustained behavioral effects of lithium exposure during early development in zebrafish: involvement of the Wnt-beta-catenin signaling pathway. *Progress in neuro-psychopharmacology & biological psychiatry*. 2014;55:101-8.
